# Use of a stent to treat colonic stenosis secondary to acute pancreatitis: A case report

**DOI:** 10.1016/j.ijscr.2019.06.065

**Published:** 2019-07-08

**Authors:** Jiro Kimura, Alan Kawarai Lefor, Shota Fukai, Tadao Kubota

**Affiliations:** a Department of Surgery, Tokyo Bay Urayasu Ichikawa Medical Center, Chiba, Japan; b Department of Surgery, Jichi Medical University, Tochigi, Japan

**Keywords:** CT, computed tomography, Colonic stent, Colonic stenosis, Acute pancreatitis

## Abstract

**Introduction:**

We report a patient for whom a colonic stent was successfully used to treat colonic stenosis secondary to acute pancreatitis.

**Presentation of case:**

A 70-year-old male presented with epigastric pain. A choledocholith and bile duct dilatation were found on abdominal computed tomography scan. Endoscopic retrograde cholangiopancreatography was performed and a common bile duct stent was placed. Ten hours after stent placement, severe epigastric pain developed. Contrast enhanced abdominal computed tomography scan revealed increased density of fat tissue around the pancreas. He was diagnosed with severe acute pancreatitis and treated in the intensive care unit. On the twenty-eighth hospital day, he vomited. His abdomen was distended and tender to palpation. Contrast enhanced abdominal computed tomography scan revealed stenosis of the descending colon and proximally dilated colon and small bowel. He was diagnosed with colonic stenosis secondary to acute pancreatitis. A colonic stent was placed in the descending colon. Eight months after this episode, elective subtotal colectomy was performed. The postoperative course was unremarkable.

**Discussion:**

The overall documented leak rate for segmental colectomy with or without on-table lavage following large bowel obstruction is about 4%. In addition, in the acute phase of severe acute pancreatitis, inflammation makes surgery difficult. Use of enteric stents in patients with pancreatitis could be used as a temporizing measure until the inflammation and obstruction improve.

**Conclusion:**

Colonic stenting is useful as a bridge to surgery in the management of large bowel obstruction.

## Highlights

• A colonic stent was used to treat colonic stenosis secondary to acute pancreatitis.
• Surgery should be postponed until the inflammation and obstruction improve.
• Use of enteric stents could be used as a temporizing measure before surgery.

## 1 Introduction

Over the last two decades, self-expanding enteric stents have gained popularity and have the potential to treat patients with strictures, obstruction, fistulae, and perforations of the gastrointestinal tract. For stenosis, they are mainly placed for malignant lesions. We report a patient for whom a colonic stent was successful for colonic stenosis secondary to acute pancreatitis. This work has been reported in line with the SCARE criteria [1].

## 2 Presentation of case

**Fig. 1:**
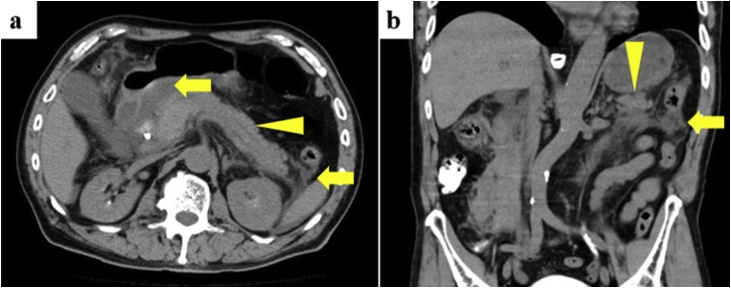
Contrast enhanced abdominal computed tomography scan revealed increased density of fat tissue (arrow) around the pancreas (arrowhead). **a** axial view, **b** coronal view.

A 70-year-old male presented with a one-day history of epigastric pain. His vital signs were normal. Abdominal examination showed epigastric tenderness to palpation and a positive Murphy sign. Laboratory studies revealed elevated serum total bilirubin (1.95 mg/dL). A choledocholith and bile duct dilatation were found on abdominal computed tomography (CT) scan. He was diagnosed with choledocholithiasis. Endoscopic retrograde cholangiopancreatography showed a choledocholith and a common bile duct stent was placed. Ten hours after stent placement, he developed severe epigastric pain. Abdominal examination showed epigastric tenderness on palpation without rebound tenderness. Laboratory studies showed an elevated serum lipase level (3345 IU/L). Contrast enhanced abdominal CT scan revealed increased density of fat tissue around the pancreas (Fig. 1). His age (70 years old), hematocrit (48.0%), and Glasgow Coma Scale = 14 (due to confusion) resulted in eight points on the Acute Physiology, Age, Chronic Health Evaluation II score. He was diagnosed with severe acute pancreatitis and treated in the intensive care unit.

**Fig. 2:**
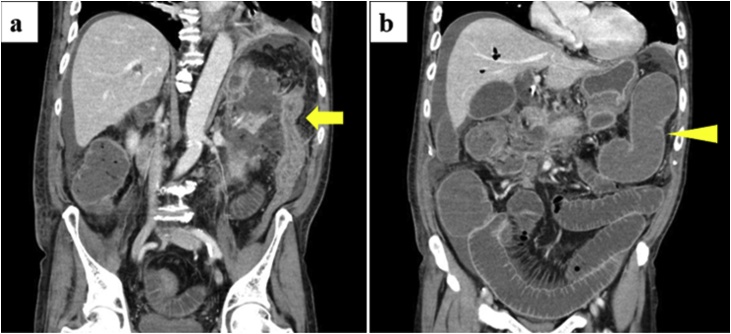
Contrast enhanced abdominal computed tomography scan revealed **a** stenosis in the descending colon near the splenic flexure region (arrow); **b** proximal dilated colon (arrowhead) and small bowel.

**Fig. 3:**
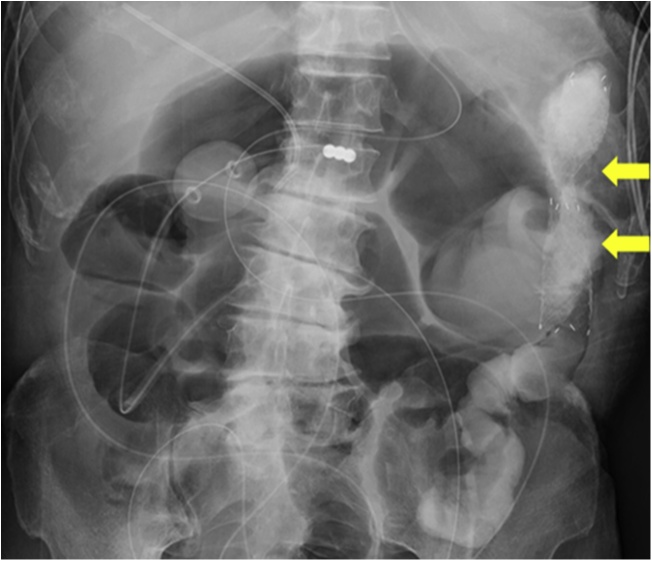
A colonic stent (arrow) was placed in the descending colon.

On the twenty-eighth hospital day, he vomited two times. His abdomen was soft, distended and slightly tender to palpation. Contrast enhanced abdominal CT scan revealed stenosis of the descending colon near the splenic flexure and proximal dilated colon and small bowel (Fig. 2). Colonoscopy showed stenosis without mucosal abnormalities in the descending colon. He was diagnosed with colonic stenosis secondary to acute pancreatitis. A naso-jejunal tube was placed. However, his symptoms did not improve. Operative management was difficult because of severe peri-colic inflammation. Therefore, a colonic stent was placed in the descending colon (Fig. 3). Placement was successful and he was discharged 11 days later.

**Fig. 4:**
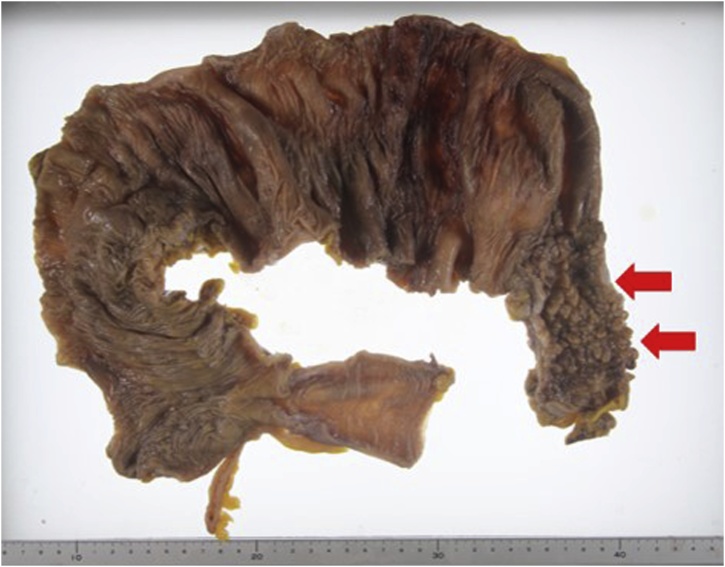
The specimen showed stenosis of the descending colon without malignancy.

Eight months after this episode of severe acute pancreatitis elective subtotal colectomy was performed, instead of a left colectomy, for a safer anastomosis. There were few intraperitoneal adhesions because the inflammation had resolved over eight months, and the operation proceeded without difficulty. The postoperative course was unremarkable and he was discharged without complications. The specimen showed stenosis of the descending colon without malignancy (Fig. 4).

## 3 Discussion

Colonic complications from acute pancreatitis are rare and are associated with mortality and morbidity [2,3]. They include bowel obstruction, paralytic ileus, bowel necrosis, fistula, and perforation [4]. The exact frequency of these complications has not been defined. A retrospective review of 296 patients revealed that 6.1% developed colonic complications. Only one patient had colonic stenosis [5].

Over　the　last　two　decades,　self-expanding　enteric　stents　have　gained in popularity and have therapeutic utility for strictures, obstruction, fistulae, and perforations of the gastrointestinal tract [6,7]. Stents have been mainly used to treat obstructing malignant lesions [8]. Their use in benign disease remains a controversial area [9].

Technical advances have allowed the use of stents in the splenic flexure [10], but there is no reported use in patients with complications associated with pancreatitis. Experience with other benign diseases suggests that insertion of a self-expanding stent is a safe procedure but surgery is required in a substantial proportion of patients due to primary or secondary failure [9]. To the best of our knowledge, the present patient is the first report of stenting for colonic stenosis secondary to acute pancreatitis.

The overall documented leak rate for segmental colectomy with or without on-table lavage following large bowel obstruction is about 4% [[11], [12], [13]]. In addition, in the acute phase of severe acute pancreatitis, inflammation makes surgery difficult. Use of stents in patients with pancreatitis could be used as a temporizing measure until the inflammation and obstruction improve. It is reasonable to use the stent as a “bridge to surgery” for patients with acute pancreatitis and colonic obstruction.

## 4 Conclusion

Large bowel obstruction is a rare complication of acute pancreatitis but one that clinicians should be aware of due to its high morbidity. Colonic stenting is useful as a “bridge to surgery” in the management of large bowel obstruction. However, it needs further evaluation in this benign setting.

## Funding

Authors had no sources of funding.

## Ethical approval

IRB/Ethics Committee ruled that approval was not required for this study.

## Consent

Written informed consent was obtained from the patient for publication of this case report and accompanying images. A copy of the written consent is available for review by the Editor-in-Chief of this journal on request.

## Author contribution

The work presented was carried out in collaboration between all authors. JK, AKL, SF and TK defined the research theme, discussed analyses and approved the final version to be published. JK analyzed the data, interpreted the results and wrote the paper.

## Registration of research studies

There is no need to register because it is a case report.

## Guarantor

Jiro Kimura.

## Provenance and peer review

Not commissioned, externally peer-reviewed.

## Declaration of Competing Interest

All authors have no conflict of interest.
